# Evaluation of performance of the GENECUBE assay for rapid molecular identification of *Staphylococcus aureus* and methicillin resistance in positive blood culture medium

**DOI:** 10.1371/journal.pone.0219819

**Published:** 2019-07-16

**Authors:** Yukio Hida, Keiichi Uemura, Hiroyasu Sugimoto, Yosuke Kawashima, Norito Koyanagi, Shigeyuki Notake, Yusaku Akashi, Shohei Sakaguchi, Hideki Kimura, Hiromichi Suzuki

**Affiliations:** 1 Department of Clinical Laboratory, University of Fukui Hospital, Yoshida-gun, Fukui, Japan; 2 Department of Clinical Laboratory, Chutoen General Medical Center, Kakegawa, Shizuoka, Japan; 3 Tsuruga Institute of Biotechnology, TOYOBO Co., Ltd., Tsuruga, Fukui, Japan; 4 Diagnostic System Department, TOYOBO Co., Ltd., Osaka, Osaka, Japan; 5 Department of Clinical Laboratory, Tsukuba Medical Center Hospital, Tsukuba, Ibaraki, Japan; 6 Department of Clinical Laboratory Medicine, Tsukuba Medical Center Hospital, Tsukuba, Ibaraki, Japan; 7 Division of Infectious Disease, Department of Medicine, Tsukuba Medical Center Hospital, Tsukuba, Ibaraki, Japan; Defense Threat Reduction Agency, UNITED STATES

## Abstract

Rapid identification of causative agents from positive blood culture media is a prerequisite for the timely targeted treatment of patients with sepsis. The GENECUBE (TOYOBO Co., Ltd.) is a novel, fully-automated gene analyzer that can purify DNAs and amplify target DNAs. In this study, we evaluated the ability of two newly developed GENECUBE assays to directly detect the *nuc* and *mecA* genes in blood culture medium; *nuc* is specific to *Staphylococcus aureus*, and *mecA* indicates methicillin resistance. We examined 263 positive blood culture samples taken at three hospitals from patients suspected of having staphylococcal bacteremia. The results were then compared with those obtained using matrix-assisted laser desorption/ionization time-of-flight mass spectrometry, antimicrobial susceptibility testing (Microscan system or Dry-plate EIKEN), and sequencing analysis. The GENECUBE assays had sensitivity and specificity of 100% in detecting both *S*. *aureus* and methicillin resistance in positive blood culture. The turnaround time of the examination was evaluated for 36 positive blood culture samples. The time between the initiation of incubation and completion of the GENECUBE examination was 23 h (interquartile range: IQR 21–37 h); the time between reporting of Gram stain examination and completion of the GENECUBE examination was 52 min (IQR 48–62 min). These findings show that the GENECUBE assays significantly reduce the assay time with no loss of sensitivity or specificity.

## Introduction

Staphylococci are one of the main causes of bloodstream infections (BSIs) [1,2]. BSIs caused by *Staphylococcus aureus* are associated with high mortality [3,4] and delay in effective antimicrobial therapy is associated with poor prognosis [5]. Penicillinase-resistant semisynthetic penicillins and first-generation cephalosporins have been used for the treatment of methicillin-susceptible *S*. *aureus* (MSSA) bacteremia with nearly equal effectiveness [6,7]. However, methicillin-resistant *S*. *aureus* (MRSA) is common worldwide [8] and the resistance rates for oxacillin were reported to be 63.2% in *S*. *aureus* and 76.2% in coagulase-negative staphylococci in the latest study in China [9]. MRSA have acquired a staphylococcal cassette chromosome *mec* (SCC*mec*) element carrying the *mec* gene, which encodes a specific penicillin-binding protein (PBP2a), causing resistance to beta-lactam antibiotics [10]. Vancomycin is effective against both methicillin-susceptible (MS) and methicillin-resistant (MR) strains, but its clinical efficacy toward MSSA can be lower than that of beta-lactam antimicrobial agents [11,12].

Information about methicillin resistance is therefore crucial for treatment of *S*. *aureus* infection. However, bacterial culture and conventional antimicrobial susceptibility testing requires at least 2 days. Recently, molecular approaches have been developed for the rapid diagnosis of BSIs using blood cultures [13]. Considering methicillin resistance, eleven types of SCC*mec* (I to XI) carrying different *mec* gene complexes have been reported and all except type XI include the gene *mecA* [10]. Thus, the detection of *mecA* has been recognized as efficient for the rapid identification of methicillin resistance. Conventionally, molecular analysis for the detection of staphylococci and methicillin resistance has been performed manually with electrophoresis [14], taking hours to obtain the results and resulting in a heavy workload for technicians. In the last 10 years, several assays with automated molecular identification systems [13] such as the BD MAX system [15–17], GeneXpert system [18], Verigene system [19–20] and FilmArray system [21] are now commercially available. These examinations are performed automatically with positive blood culture samples and clinical effectiveness has been reported [22–25]. Hands-on time is just a few minutes, but processing requires at least 1 h.

GENECUBE (TOYOBO Co., Ltd., Osaka, Japan) is a fully automated rapid genetic analyzer capable of extracting nucleic acids from biological material, preparing reaction mixtures, and amplifying a target gene by PCR. This device can handle a maximum of eight samples at once and analyze up to four items at the same time. The amplified target DNA is hybridized with a fluorescently-labeled oligonucleotide (Qprobe). Upon binding to the target DNA, the fluorescence of the Qprobe is quenched by the guanine bases in the target. However, the fluorescence reappears as the Qprobe disassociates from the melting target [26]. By detecting this change in fluorescence intensity, target genes are detected. Data are automatically obtained on the GENECUBE monitor display after completion of the examination. In the GENECUBE system, purification mode, amplification mode or both modes can be selected for each assay; amplification mode is used for PCR of purified samples or direct PCR of prepared samples. Currently, assays for *Mycobacterium tuberculosis* [27], *M*. *avium*, *M*. *intracellulare*, *Neisseria gonorrhoeae* [28], *Chlamydia trachomatis* [28], and *Mycoplasma pneumoniae* [29,30] have been released in Japan.

The GENECUBE nuc assay can identify *S*. *aureus* by targeting the *S*. *aureus*-specific *nuc* gene [31]. The GENECUBE mecA assay can detect the *mecA* gene. Both assays were designed for direct PCR from biological material without purification and the examinations can be performed automatically in about 35 min after preparation using positive blood culture samples (Table 1).

**Table 1:** Main characteristics of GENECUBE and other rapid molecular assays.

	GENECUBE nuc and GENECUBE mecA	Xpert MRSA/SA BC	BD Max StaphSR	Verigene BC-GP	FilmArray Blood Culture Identification Panel
Method	PCR	Real-time PCR	Real-time PCR	Hybridization without nucleic acid amplification, microarray technology	Nested multiplex PCR, microarray technology
Hands-on time (min per sample)	3	1	1	5	2
Approximate processing time (min)	35	60	120	150	60
Maximum number of test samples	8 per system	2 to 80 per system ^a^	24 per system	1 per processor	1 per processor
Sensitivity /specificity^b^	100%/100%^c^	98.1% /99.6%^c^	97.9–100% /98.1–100%^c^	99.7–100% / 97.5–99.5% ^c^	98.4%/98.3% ^c^

^a^ Maximum number of test samples differs by module type.

^b^ Sensitivity and specificity for the detection of methicillin resistance.

^c^ References [15–21].

Because of their easy preparation and short examination time, GENECUBE assays are expected to have clinical utility for the rapid diagnosis of *S*. *aureus* and methicillin resistance. However, their performance has not been clinically validated. Therefore, here, we performed a multicenter study to evaluate the ability of GENECUBE assays to detect *S*. *aureus* and methicillin resistance in 263 blood culture samples and compared the results with those from the standard culture method as a reference.

## Materials and methods

### Study design (samples and strains)

This study was performed to evaluate the clinical performance of GENECUBE examinations in detection of *S*. *aureus* and methicillin resistance in positive blood culture samples. Bacterial identification was performed by matrix-assisted laser desorption/ionization time-of-flight mass spectrometry (MALDI-TOF MS; Microflex LT, Bruker Daltonics, Bremen, Germany) and methicillin resistance was determined by antimicrobial susceptibility testing and detection of the *mecA* gene.

Fresh and frozen blood culture samples (−80°C) were obtained from three hospitals (University of Fukui Hospital [UFH], Chutoen General Medical Center [CGMC], and Tsukuba Medical Center Hospital [TMCH]). For fresh blood culture samples, evaluations were performed immediately when Gram-positive cocci in clusters were confirmed by Gram-stain examination of the samples; these samples were obtained between August 2017 and August 2018. The frozen blood culture samples were preserved between July 2016 and July 2017 at TMCH. If multiple positive blood culture samples were obtained from a single patient, we performed the GENECUBE examination using only one sample.

Ethical approval to use the clinical blood cultures was granted by the Review Board Committee of University of Fukui Hospital (approval number: 20170114), the Review Board Committee of Chutoen General Medical Center (approval number: 2017-C61), and the Review Board Committee of Tsukuba Medical Center Hospital (approval number: 2017–029).

### Identification and antimicrobial testing

In each of the three hospitals, blood culture was performed using in-house blood culture machines (UFH and TMCH used a BACTEC FX [BD, Franklin Lakes, NJ, USA]; CGMC used a Versa TREK [Kohjin Bio Co., Ltd., Saitama, Japan]). Two or three pairs of culture bottles for aerobes or anaerobes were incubated in a blood culture system after inoculation with blood drawn from the patients at each hospital. When a positive signal was obtained from the blood culture system and bacterial growth was noted with Gram-positive cocci in clusters, the culture sample was inoculated onto both blood agar and MRSA-selective agar plates, which were then cultured overnight in an incubator. Isolates were classified as staphylococci based on colony morphology, Gram staining and biochemical test results, and were further verified by MALDI-TOF MS. The Bruker Biotyper 3.1 software and library were used for spectral analyses. According to the manufacturer’s instructions, scores of >2.0 were considered to indicate identification at the species level. Antimicrobial susceptibility tests for oxacillin and cefoxitin were performed with the MicroScan WalkAway96 system (Beckman Coulter, Inc., Orange County, CA, USA) or the Dry-plate EIKEN system (EIKEN Chemical Co., Ltd., Tokyo, Japan). We set the minimum inhibitory concentration breakpoints according to recommendations of the Clinical and Laboratory Standards Institute [32].

### PCR and sequencing

Using bacterial colonies on primary culture plates, the presence of a *mecA* gene was identified by conventional PCR and verified by DNA sequencing [33]. Colonies were suspended in 100 μL of sterile distilled water and heated at 100°C for 5 min. Then, the suspension was centrifuged at 15,000 rpm for 1 min. The primers used for PCR and DNA sequencing analysis are listed in Table 2. PCR was performed in a 25 μL mixture containing 1× Buffer for KOD -Plus- ver. 2, 1.5 mM MgSO_4_, 200 μM of each deoxynucleoside triphosphate, 0.5 U of KOD -Plus- (TOYOBO Co., Ltd.), 0.2 μM of *mecA* primers, and 1 μL of isolated colony supernatant. The thermocycling conditions in the Gene Atlas thermocycler (ASTEC, Fukuoka, Japan) were: 94°C for 2 min, followed by 30 cycles of 98°C for 10 s, 55°C for 30 s and 68°C for 30 s.

**Table 2:** Primers used for conventional PCR and direct sequencing.

Primer	Sequence (5ʹ–3ʹ)	Amplicon size (bp)
*mecA*-1	AAAATCGATGGTAAAGGTTGGC	533
*mecA*-2	AGTTCTGCAGTACCGGATTTGC	

PCR, polymerase chain reaction.

The amplified PCR products were purified using a QIAquick PCR Purification Kit (QIAGEN Sciences, Chatsworth, CA, USA), labeled with a BigDye Terminator 3.1 Cycle Sequencing Kit (Applied Biosystems, Foster, CA, USA), and applied to a 3730xl DNA Analyzer (Thermo Fisher Scientific, Waltham, MA, USA).

### The GENECUBE assay

First, we performed Gram stain examination of blood cultures. When Gram-positive cocci in clusters were seen, the GENECUBE assay was performed. Prepared samples were analyzed using the GENECUBE system according to the manufacturer’s instructions. PCR was performed using specimens prepared by diluting blood culture medium with alkaline lysis solution. For the analysis of *nuc* and *mecA* genes in the blood culture medium, the lysed specimens were used directly without purification in automated GENECUBE examination including a PCR and a melting point analysis. The PCR conditions were: denaturation at 94°C for 30 s, and 60 cycles of 97°C for 1 s, 58°C for 3 s and 63°C for 5 s. The PCR products were subjected to a melting point analysis, the conditions of which were: 94°C for 30 s and 39°C for 30 s, followed by heating from 40°C to 75°C in increments of 0.09°C/s. We performed all procedures on-site at the hospital laboratory, and those performing the assays were blinded to the culture results.

### Determination of the limits of detection (LODs), PCR inhibition by blood culture media and turnaround time of GENECUBE examination

As basic data, we examined the LODs of GENECUBE nuc and GENECUBE mecA for the detection of the *nuc* and *mecA* genes respectively, and investigated inhibition of PCR by blood culture media. We also investigated the turnaround time of the GENECUBE examination.

The LODs of the reagents were determined using AMPLIRUN STAPHYLOCOCCUS AUREUS (*mecA*+) DNA CONTROL (Vircell SL, Granada, Spain) as a target gene. The genomic DNA was diluted twofold in series (12.5–1.56 copies/μL) with 10 mM Tris-HCl (pH 7.5). Four dilutions (4 μL each) were tested in replicates of eight at each concentration in the GENECUBE nuc and GENECUBE mecA assays. The LODs were estimated as the lowest concentration of genomic DNA where the positivity rate was 100%.

To test the influence of blood culture media on the DNA amplification, PCR with and without the genomic DNA was conducted with different dilutions of blood culture medium. Eight kinds of blood culture media (BacT/ALERT series [FA Plus, FN Plus, PF Plus; bioMérieux, Marcy l'Etoile, France], BACTEC series [Aerobic/F, Anaerobic/F, Peds Plus/F] and Versa TREK series [REDOX 1, REDOX 2]) were diluted 50-, 100-, 200- and 300-fold with water or water containing the genomic DNA at 12.5 copies/μL. Four positive dilutions and four negative dilutions of each medium were tested in quadruplicate using GENECUBE nuc and GENECUBE mecA assays. We estimated the lowest dilution where detection of the target or internal control was not inhibited.

The GENECUBE examinations are performed with positive blood culture samples after Gram stain examination. Therefore, for investigation of the turnaround time of GENECUBE examination, the time of initiation of incubation of blood culture bottles, the reporting time of Gram stain examination, and the completion time of the GENECUBE examination were recorded at TMCH between February 2018 and August 2018.

### Statistical analyses

The GENECUBE assay results were compared with each result of MALDI-TOF mass spectrometry, antimicrobial susceptibility testing and sequencing analysis for *mecA*. The sensitivity, specificity, positive predictive value and negative predictive value were calculated from routine 2×2 result tables. The 95% confidence intervals (CIs) were calculated by the method of Clopper and Pearson using the online calculator at http://statpages.info/confint.htm.

## Results

### LODs of the GENECUBE assays

Both assays detected all replicates at ≥12.5 copies/test (Table 3); 11/16 (68.8%) were positive at 6.25 copies/test with GENECUBE nuc, and 13/16 (81.3%) were positive with GENECUBE mecA. Based on these results, the LODs of GENECUBE nuc and GENECUBE mecA were both estimated to be 12.5 copies/test.

**Table 3:** Detection limits of GENECUBE nuc and GENECUBE mecA.

Kit	Copies/test	Numbers of positives	Positivity rate (%)
GENECUBE nuc	50	16/16	100
	25	16/16	100
	12.5	16/16	100
	6.25	11/16	68.8
GENECUBE mecA	50	16/16	100
	25	16/16	100
	12.5	16/16	100
	6.25	13/16	81.3

### PCR inhibition by blood culture media

The results of tests of PCR inhibition by blood culture media are shown in Table 4. In the BacT/ALERT series, PCR inhibition was shown at ≤100-fold dilution in the GENECUBE nuc and GENECUBE mecA assays. In the BACTEC series, ≥100-fold dilution was required for GENECUBE nuc and 300-fold dilution for GENECUBE mecA to avoid PCR inhibition. In the VersaTREK series, no inhibition was shown in the *nuc* assay at ≥50-fold dilution, but the *mecA* assay detected only 3/4 replicates at 100-fold dilution with Versa TREK REDOX2.

**Table 4:** Numbers of positive replicates in the GENECUBE nuc and GENECUBE mecA assays in diluted blood culture media.

			GENECUBE nuc		GENECUBE mecA	
Blood culture medium	Dilution rate (-fold)	Genomic DNA	target (*nuc*)	IC	target (*mecA*)	IC
BacT/ALERT FA Plus	50	+	0/4	0/4	0/4	0/4
		−	-	0/4	-	0/4
	100	+	0/4	0/4	0/4	0/4
		−	-	0/4	-	0/4
	200	+	4/4	4/4	4/4	4/4
		−	-	4/4	-	4/4
	300	+	4/4	4/4	4/4	4/4
		−	-	4/4	-	4/4
BacT/ALERT FN Plus	50	+	0/4	0/4	0/4	0/4
		−	-	0/4	-	0/4
	100	+	0/4	0/4	0/4	0/4
		−	-	0/4	-	0/4
	200	+	4/4	4/4	4/4	4/4
		−	-	4/4	-	4/4
	300	+	4/4	4/4	4/4	4/4
		−	-	4/4	-	4/4
BacT/ALERT PF Plus	50	+	0/4	0/4	0/4	0/4
		−	-	0/4	-	0/4
	100	+	0/4	0/4	4/4	0/4
		−	-	0/4	-	0/4
	200	+	4/4	4/4	4/4	4/4
		−	-	4/4	-	4/4
	300	+	4/4	4/4	4/4	4/4
		−	-	4/4	-	4/4
BACTEC Plus Aerobic/F Culture Vilas	50	+	0/4	0/4	0/4	0/4
		−	-	0/4	-	0/4
	100	+	4/4	4/4	4/4	4/4
		−	-	4/4	-	4/4
	200	+	4/4	4/4	4/4	4/4
		−	-	4/4	-	3/4
	300	+	4/4	4/4	4/4	4/4
		−	-	4/4	-	4/4
BACTEC Plus Anaerobic/F Culture Vilas	50	+	0/4	0/4	0/4	0/4
		−	-	1/4	-	0/4
	100	+	4/4	4/4	4/4	4/4
		−	-	4/4	-	4/4
	200	+	4/4	4/4	4/4	3/4
		−	-	4/4	-	4/4
	300	+	4/4	4/4	4/4	4/4
		−	-	4/4	-	4/4
BACTEC Peds Plus/F Culture Vilas	50	+	4/4	4/4	4/4	4/4
		−	-	4/4	-	4/4
	100	+	4/4	4/4	4/4	4/4
		−	-	4/4	-	4/4
	200	+	4/4	4/4	4/4	4/4
		−	-	4/4	-	4/4
	300	+	4/4	4/4	4/4	4/4
		−	-	4/4	-	4/4
VersaTREK REDOX 1	50	+	4/4	4/4	4/4	4/4
		−	-	4/4	-	4/4
	100	+	4/4	4/4	4/4	4/4
		−	-	4/4	-	4/4
	200	+	4/4	4/4	4/4	4/4
		−	-	4/4	-	4/4
	300	+	4/4	4/4	4/4	4/4
		−	-	4/4	-	4/4
VersaTREK REDOX 2	50	+	4/4	4/4	4/4	4/4
		−	-	4/4	-	4/4
	100	+	4/4	4/4	4/4	3/4
		−	-	4/4	-	3/4
	200	+	4/4	4/4	4/4	4/4
		−	-	4/4	-	4/4
	300	+	4/4	4/4	4/4	4/4
		−	-	4/4	-	4/4

IC, internal control; +, positive; -, negative.

### Agreement between the results of the GENECUBE assays and microbiological assays

A total of 263 blood culture samples (frozen blood culture media: 48 bottles; fresh blood culture media: 215 bottles) obtained at three hospitals (UFH: 90 bottles; CGMC: 72 bottles; TMCH: 101 bottles) were evaluated in this study. During the evaluation, no PCR inhibition was observed in the two GENECUBE assays for either frozen or fresh blood culture media. The conditions of the samples did not affect the results.

The results obtained from conventional microbiological analysis and the GENECUBE examinations are summarized in Table 5. Among the 263 blood culture samples tested, 102 were positive for *S*. *aureus* (44 MRSA, 56 MSSA, and two mixtures of staphylococcal species [MSSA + MR coagulase-negative staphylococci [MRCoNS], and MSSA + MS coagulase-negative staphylococci [MSCoNS]]). The 102 samples were classified as *nuc* gene-positive based on the detection of *S*. *aureus* in culture. On comparison with the results from MALDI TOF-MS, the sensitivity of detection of *S*. *aureus* in blood culture media was 100% in the GENECUBE nuc assay (95% CI: 98.2%–100%). In the remaining 161 blood culture samples, we detected a wide variety of CoNS (*S*. *epidermidis*, 96 strains; *S*. *lugdunensis*, one strain; other CoNS, 58 strains; three mixtures of two staphylococcal species [MS *S*. *epidermidis* + MR *S*. *epidermidis*, MR *S*. *epidermidis* + MR *S*. *capitis*, and MS *S*. *epidermidis* + MS *S*. *hominis*]), and three strains of non-*Staphylococcus* species (*Aerococcus urinae*, one strain; *Micrococcus* spp., two strains). The *nuc* gene was not detected in any of these 161 blood culture samples.

**Table 5:** Agreement between results from the GENECUBE and microbiological assays.

	Results of GENECUBE assays						
	*nuc*		*mecA*		Sensitivity (%) ^c^	Specificity (%)	PPV NPV (%)
	Positive	Negative	Positive	Negative			
MALDI TOF-MS ^a^							
*S*. *aureus*	100	0			100 (98.1–100)^d^	100 (98.8–100)^d^	100 (98.1–100) 100 (98.8–100)
Others ^b^	0	161					
*S*. *aureus* + Others	2	0					
Antimicrobial test							
MR			156	0	100 (98.8–100)^e^	100 (98.2–100)^e^	100 (98.8–100) 100 (98.2–100)
MS			0	105			
MS+MR			2	0			
Sequencing analysis of *mecA* gene							
Positive			158	0	100 (98.8–100)	100 (98.2–100)	100 (98.8–100) 100 (98.2–100)
Negative			0	105			

*S*. *aureus*, *Staphylococcus aureus*; MR, methicillin-resistant; MS, methicillin-sensitive; MALDI TOF-MS, matrix-assisted laser desorption/ionization time-of-flight mass spectrometry; PPV, positive predictive value; NPV, negative predictive value.

^a^ The Bruker Biotyper 3.1 software program and library (Bruker Daltonics, Bremen, Germany) were used for spectral analyses. According to the manufacturer’s instructions, scores of >2.0 were considered to indicate identification at the species level.

^b^ Included coagulase-negative staphylococci (157), *Staphylococcus lugdunensis* (1), *Aerococcus urinae* (1) and *Micrococcus* spp. (2).

^c^ Data in parentheses are 95% confidence intervals.

^d^ Sensitivity or specificity for detecting *S*. *aureus* in blood culture media.

^e^ Sensitivity or specificity for detecting the *mecA* gene in blood culture media.

Regarding methicillin resistance, *mecA* was positive in 158 blood culture samples according to GENECUBE examination, and MR staphylococci were isolated from all 158 blood culture samples (44 MRSA, 112 MRCoNS, one MSSA + MRCoNS, and one MSCoNS + MRCoNS). Regarding MR staphylococci, the sensitivity of detection of the *mecA* gene in blood culture medium was 100% (95% CI: 98.8%–100%). In the remaining 105 blood culture samples, MR staphylococci were not isolated, and the *mecA* gene was not detected in any of the 105 blood culture samples. The overall agreement between the results of the GENECUBE assay for blood culture medium and sequencing analysis of isolated strains was 100% (95% CI: 98.6%–100%).

### Turnaround time of GENECUBE examination

Mean time from the initiation of incubation to completion of the GENECUBE examination (N = 36) was 23.0 h (IQR 20.8–37.0). Time from reporting of the Gram stain examination to completion of the GENECUBE examination was 52.0 min (IQR 48.0–61.8).

## Discussion

From this basic study, the LODs of the GENECUBE nuc and GENECUBE mecA assays were estimated to be 12.5 copies/test, and 324-fold dilution of blood culture media with lysis solution for examination with GENECUBE nuc and GENECUBE mecA was deemed appropriate for preventing PCR inhibition by blood culture media. The coefficients of correlation for the log colony-forming units (CFU) and *nuc* gene copy numbers were reported to range from 0.98 to 1.00 [34]. Based on these data, GENECUBE nuc and GENECUBE mecA are considered to detect *nuc* and *mecA* genes in positive blood culture samples if the bacterial concentration exceeds 1×10^6^ CFU/mL. The bacterial concentration at the time of blood culture positivity is known to be between 2×10^7^ and 7×10^9^ CFU/mL based on a previous study [35], so examination with GENECUBE nuc and GENECUBE mecA is expected to be able to detect *nuc* and *mecA* genes in positive blood culture samples.

In the current study with clinical samples, we observed that, in terms of both the sensitivity and specificity, the performance of the GENECUBE system in detection of the *nuc* and *mecA* genes in positive blood culture samples was comparable to that of conventional microbiological assay. The overall sensitivity of 100% is similar to that obtained with other rapid molecular diagnosis tools (Table 1). A mixture of two staphylococcal species (MSSA and MRCoNS) was observed in one positive blood culture sample. GENECUBE examination cannot differentiate the mixture of two staphylococcal species from MRSA, so technicians may have misidentified this case as MRSA bacteremia; careful judgment is needed, especially in suspected cases of bacterial culture contamination.

To the best of our knowledge, this is the first molecular assay with an automated molecular identification system for the detection of *S*. *aureus* and methicillin resistance without purification processes such as magnetic bead-based DNA purification or DNA purification using the Boom method [36]. The processing time for the GENECUBE nuc and GENECUBE mecA assays is shorter than that required for other molecular assays using commercially available molecular identification systems. Loop-mediated isothermal amplification can perform amplification in 45 min [37]; however, it requires a longer preparation time than the GENECUBE and other examinations. In the current study, most of the results were obtained within 1 h of Gram stain examination. Thus, clinicians could obtain information regarding methicillin-resistance in cases of staphylococcal bacteremia without delay after the report of Gram stain examination. Before cultivation, the bacterial concentration is <1 CFU/mL in more than half of patients with bacteremia [38]. Currently, there are several systems available worldwide for the detection of genes for bacterial identification and antimicrobial resistance directly from whole blood samples, such as the LightCycler SeptiFast and T2 Biosystems instruments [13, 39]. Both systems can produce the result with 6 h of processing time. However, LightCycler SeptiFast needs a long hands-on time, which is reported as 3 h [13], and the T2 Biosystems approach requires two panels to obtain results for bacterial identification and antimicrobial resistance. For the GENECUBE system, centrifugation is needed to reach the LODs for the detection of target genes directly from whole blood before cultivation. Further evaluation is required to determine whether GENECUBE analysis of centrifuged whole blood samples can be used for detection of *S*. *aureus* and methicillin resistance without an incubation step.

In addition to difficulties caused by the mixture of two staphylococcal species, several limitations associated with the GENECUBE assay must be considered. First, among the eleven types of SCC*mec* (I to XI), type XI carries the *mecC* gene which is only about 70% identical to the *mecA* gene at the DNA level [10, 40] and the GENECUBE assay cannot detect *mecC*. Second, GENECUBE examination detects the *nuc* gene in the identification of *S*. *aureus*, so *nuc*-deficient *S*. *aureus* strains will not be detected by the GENECUBE assay. While no such strains were observed in the present study, deficient strains have been reported previously [41]. We must investigate the proportion of *nuc*-deficient strains in *S*. *aureus* detected from blood cultures.

In summary, the GENECUBE system is a simple identification method and the GENECUBE nuc and GENECUBE mecA assays have been proven to be reliable for detecting both the *nuc* and *mecA* genes in positive blood culture samples. This novel method appears suitable for the rapid diagnosis of staphylococcal bacteremia.
